# Comparative analysis of bones, mites, soil chemistry, nematodes and soil micro-eukaryotes from a suspected homicide to estimate the post-mortem interval

**DOI:** 10.1038/s41598-017-18179-z

**Published:** 2018-01-08

**Authors:** Ildikó Szelecz, Sandra Lösch, Christophe V. W. Seppey, Enrique Lara, David Singer, Franziska Sorge, Joelle Tschui, M. Alejandra Perotti, Edward A. D. Mitchell

**Affiliations:** 1 Laboratory of Soil Biodiversity, University of Neuchâtel, 2000 Neuchâtel, Switzerland; 2 Department of Physical Anthropology, Institute of Forensic Medicine, University of Bern, 3007 Bern, Switzerland; 3 Real Jardín Botánico, CSIC, Plaza de Murillo 2, 28014 Madrid, Spain; 4 Institute of Legal Medicine, Goethe-University, 60596 Frankfurt/Main, Germany; 5 Department of Forensic Medicine and Imaging, Institute of Forensic Medicine, University of Bern, 3012 Bern, Switzerland; 6 Acarology Lab, Ecology and Evolutionary Biology Section, School of Biological Sciences, University of Reading, RG6 6AS Reading, United Kingdom; 7 Botanical Garden of Neuchâtel, 2000 Neuchâtel, Switzerland

## Abstract

Criminal investigations of suspected murder cases require estimating the post-mortem interval (PMI, or time after death) which is challenging for long PMIs. Here we present the case of human remains found in a Swiss forest. We have used a multidisciplinary approach involving the analysis of bones and soil samples collected beneath the remains of the head, upper and lower body and “control” samples taken a few meters away. We analysed soil chemical characteristics, mites and nematodes (by microscopy) and micro-eukaryotes (by Illumina high throughput sequencing). The PMI estimate on hair ^14^C-data via bomb peak radiocarbon dating gave a time range of 1 to 3 years before the discovery of the remains. Cluster analyses for soil chemical constituents, nematodes, mites and micro-eukaryotes revealed two clusters 1) head and upper body and 2) lower body and controls. From mite evidence, we conclude that the body was probably brought to the site after death. However, chemical analyses, nematode community analyses and the analyses of micro-eukaryotes indicate that decomposition took place at least partly on site. This study illustrates the usefulness of combining several lines of evidence for the study of homicide cases to better calibrate PMI inference tools.

## Introduction

The estimation of a post-mortem interval (PMI) or the time since death has been a research priority in forensic science for over a century since the pioneering work of Mégnin^1^ (1894), who defined the decomposition stages of corpses for the first time. Currently the minimum post-mortem interval (PMI_min_) is mainly estimated based on a medical assessment that relies on the physical changes of the dead body occurring in the first hours up to days and/or entomological evidence, a well-established method applied to periods of up to several weeks or months^2^. Although insects can be related to all decomposition stages^3,4^, the accuracy of PMI_min_ estimation decreases over time^5,6^. Additionally, methods based on tissue chemistry such as the citrate content of bones or radiocarbon dating of different human tissue can be useful^7,8^. However, until now there is no accurate PMI estimation method for human remains that have already reached the dry and remains stages. But each year a number of corpses in very advanced decomposition stages are found; obtaining a reliable PMI for these can be especially crucial if other forensic evidence is scarce. For example, in the Institute of Legal Medicine in Frankfurt am Main alone, 51 corpses with a long post-mortem interval were checked using entomological evidence in the years 2014–2016 and 20% originated from outdoor environments (V. Bernhardt personal communication). Although not very frequent, the discovery of old decomposed human corpses is a reality and new methods are therefore required for long PMI estimations.

Cadaver decomposition can be seen as a continuum of several stages: fresh, bloated, active decay, advanced decay, dry and remains^9^. The duration of each stage depends mainly on temperature, humidity and scavenger access to the cadaver^10–12^. A still limited, but increasing number of studies have focused on the effects of cadaver decomposition on the underlying soil as new venues for gathering forensic evidence. These studies monitored the changes in soil chemistry^13–16^ and the community structure of soil micro-organisms^17,18^, bacteria^19–21^, fungi^20,22,23^, testate amoebae^24,25^, nematodes^26^ and micro-arthropods^27–32^. While the relevance of mites for criminal investigations is well-established^27,30^, studies of other potential forensic indicators are still rare and comparative studies are lacking. Soil arthropods such as springtails (Collembola)^28,29^ and ants^33^ were also proven useful for PMI estimation. More recently, high-throughput sequencing (HTS) of soil organisms has been used to develop new forensic tools^24,34,35^. These methods may each provide complementary information and PMI estimations should thus become more robust when several methods are combined. Inferring forensic evidence from a decomposing cadaver is challenging and the whole picture may only appear clear when several independent lines of evidence are combined.

In the present work, five different approaches were applied to a case study, aiming to reconstruct the crime scene and the PMI. These different approaches were also combined and analysed for correlation via Multi-Factor Analysis (MFA). Human bones were found in a forest area in the Swiss midlands. Bones were examined morphologically and biochemically to determine sex and biological age of the individual and to estimate PMI. Soil samples were collected and analysed for selected chemical markers, for nematode and mite diversity (based on morphology), and micro-eukaryotes (using high throughput DNA amplicon sequencing, i.e. metabarcoding) to assess possible differences in community structure. Due to legal reasons, some details about the finding must be kept under nondisclosure.

## Results

### Bone and hair analyses

**Figure 1:**
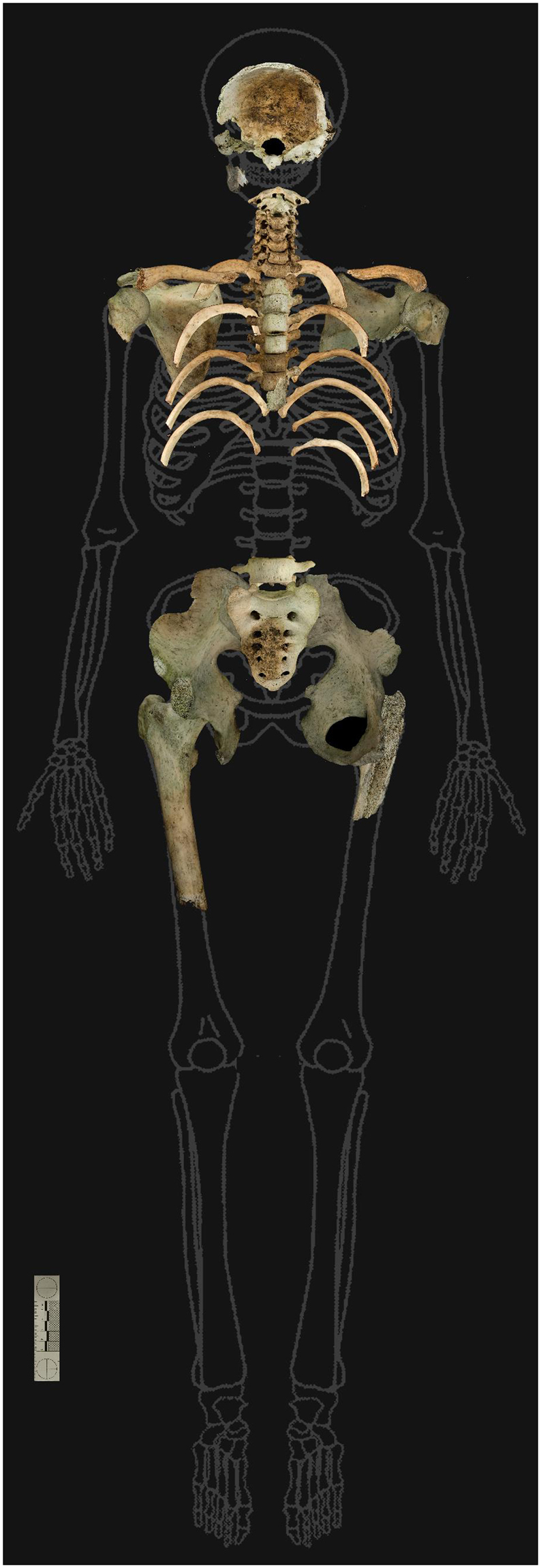
Identified bones from human remains (burnt fragments are not shown) found in a forest area in Switzerland.

The preservation and the representation of the identifiable skeletal elements were low due to signs of thermal destruction caused by burning (Fig. 1). Particularly affected by the fire were the limbs including hands and feet. Parts of the pelvis and skull were present allowing a sex determination as male. An age at death was estimated to be between 18 to 25 years. The ^14^C in the collagen of the femur was integrated approximately between 10–12 years before (1 sigma) the remains were found. The ^14^C in the hair was integrated approximately in the last 3 years before (1 sigma) the investigation.

### Chemical analyses

**Figure 2:**
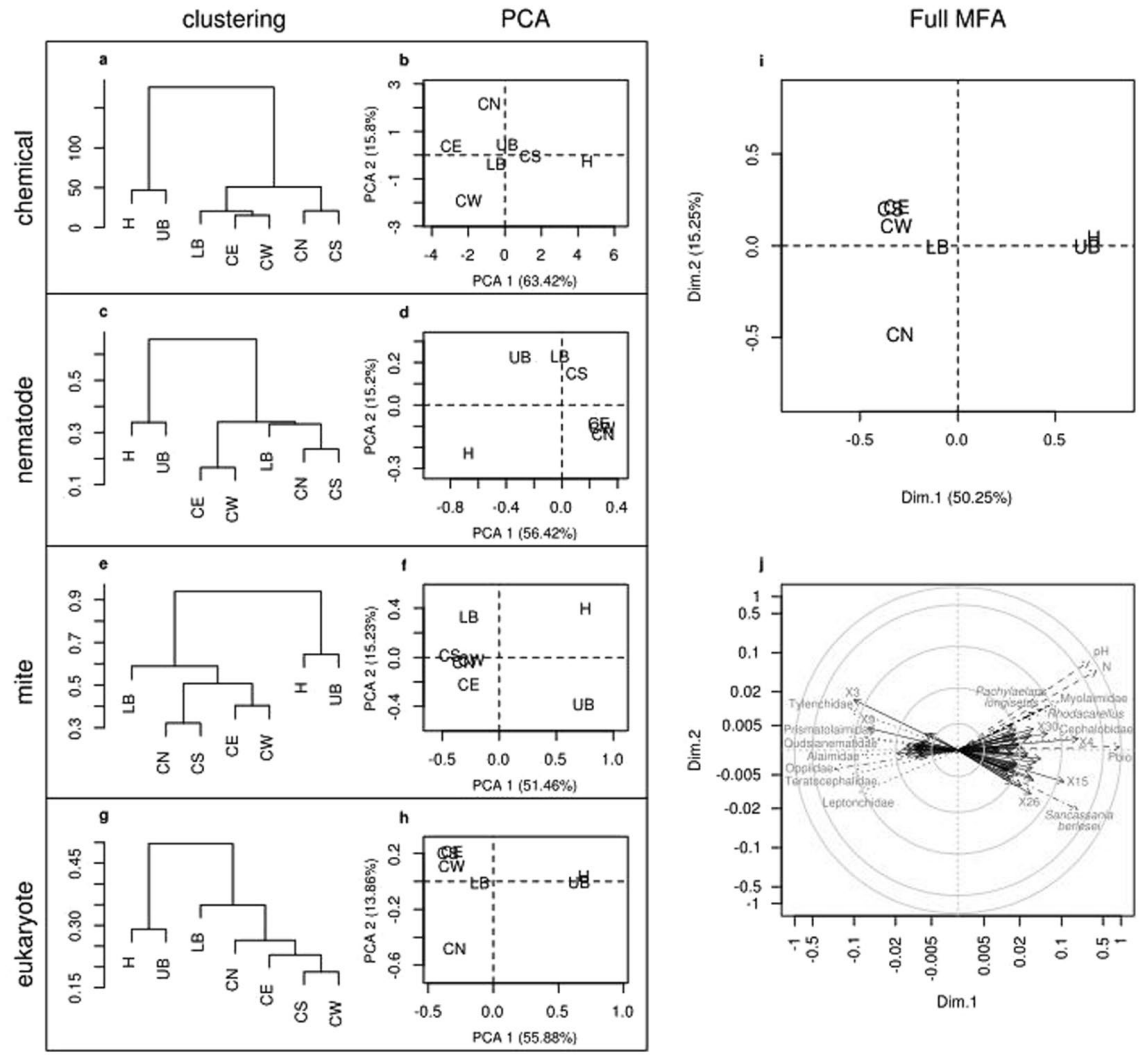
Multiple factor analysis (MFA) on chemical variables and community matrices of nematodes, mites and of micro-eukaryotes from soil samples taken beneath human remains and controls in a forest area in Switzerland. On the left part the projections of the samples according to the clustering (**a,c,e,g**) and the PCA (**b,d,f,h**) calculated on chemical variables (**a**,**b**), nematode families (**c**,**d**), mites taxa (**e**,**f**) and micro-eukaryotic OTUs (**g,h**) are represented. On the right part of the figure the projection of the samples according to the global analysis (**i)** and the correlation circle (**j)** of the most characteristic variable (P < 0.05) of the first and second dimensions are shown. The four variable types are represented with bold italic font and dashed arrows for chemical variables, bold font and dotted arrows for nematode families, italic font and dot dash arrows for mite taxa and normal font and plain arrows for micro-eukaryotic OTUs. In order to improve readability, only names of variables with a distance between the origin and their coordinate higher than 0.05 were shown. The correlation circle is shown on a log scale because of the difference between coordinates from eukaryotic and chemical data.

Soil bioavailable P content was higher beneath the head (192.54 µg/g) and upper body (147.99 µg/g) than under the lower body (LB) and control samples (ranging from 17.52 µg/g in control West to 65.87 µg/g in control South, Table 1). Total N was at least twice as high under the head compared to other samples (Table 1). Other soil chemical markers (pH, NH_4_^+^, NO_3_^−^, Mg^2+^, Ca^2+^ and C content) did not differ between the control and cadaver samples (Table 1). The cluster analysis separated the samples into two clusters: 1) head and upper body and 2) controls and lower body (Fig. 2a). In the PCA the sample from beneath the head sample was separated from the other samples (Fig. 2b).

**Table 1:** Chemical constituents in soil samples taken beneath human remains found in a forest in the Swiss Plateau at a height of approx. 400-500 m above sea level and in controls in the same area.

chemical constituents	Head	Upper Body	Lower Body	Control North	Control South	Control West	Control East
	H	UB	LB	CN	CS	CW	CE
pH	7.65	7.25	7.34	7.17	7.34	7.24	6.97
NH_4_^+^ [ug/g]	3.20	0.60	1.10	1.30	3.20	1.30	0.90
NO_3_^−^ [ug/g]	47.52	52.88	44.81	65.07	54.12	47.43	61.52
N [%]	2.86	1.15	1.09	1.19	1.35	0.54	0.61
C [%]	27.79	15.22	18.5	22.74	22.69	9.41	10.71
H [%]	2.19	1.52	0.65	0	0.99	1.17	1.23
P_bio_ [/g]	192.54	147.99	31.41	50.05	65.87	17.52	22.86
Mg^2+^ [mg/l]	0.03	0.03	0.04	0.04	0.04	0.01	0.01
Ca^2+^ [mg/l]	0.66	0.52	0.80	0.82	0.88	0.39	0.50

### Nematodes

Nematode density under the cadaver was highest beneath the head (2488 ind./100 g dry soil), lower under the upper body (1653 ind./100 g) and lowest under the lower body (1192 ind./100 g), the latter values were within the range of the control samples (982–1402 ind./100 g). In total 19 nematode families were identified (Table 2). Diversity was highest under the lower body and in the control samples (12–14 families), nine of which being present in all of these samples (Table 2). By contrast, only five and six families were present beneath the head and the upper body respectively (Table 2). Bacterial feeding Cephalobidae, Rhabditidae and Plectidae were the three most abundant families and occurred in all samples (Table 2).

**Table 2:** Density of nematode families in 100 g^−1^ dry soil.Soil samples taken beneath human remains found in a forest in the Swiss Plateau at a height of approx. 400–450 m above sea level and in controls in the same area.

nematode taxa	feeding groups	Head	Upper Body	Lower Body	Control North	Control South	Control West	Control East
		H	UB	LB	CN	CS	CW	CE
Alaimidae	bacterial feeding	0	0	36	20	14	20	84
Bastianiidae	bacterial feeding	0	0	0	20	0	10	14
Bunonematidae	bacterial feeding	0	0	12	0	0	0	14
Cephalobidae	bacterial feeding	1319	727	489	150	389	147	140
Myolaimidae	bacterial feeding	547	0	0	0	0	0	0
Plectidae	bacterial feeding	75	463	167	120	209	79	112
Prismatolaimidae	bacterial feeding	0	0	72	20	14	39	28
Rhabditidae	bacterial feeding	522	281	60	100	83	128	280
Teratocephalidae	bacterial feeding	0	66	24	120	111	157	168
Aphelenchoididae	fungal feeding	0	0	36	0	0	0	28
Leptonchidae	fungal feeding	0	99	107	50	139	128	252
Thornenematidae	omnivorous	0	0	0	20	28	10	0
Aporcelaimidae	animal predator, omnivorous	25	17	48	0	125	10	28
Qudsianematidae	animal predator, omnivorous	0	0	95	120	28	88	98
Mononchidae	animal predator	0	0	0	0	0	20	42
Diphtherophoridae	plant feeding	0	0	0	0	28	39	0
Pratylenchidae	plant feeding	0	0	12	0	14	0	0
Tylenchidae	plant feeding	0	0	24	230	181	108	112
Tylodoridae	plant feeding	0	0	12	30	28	0	0

**Figure 3:**
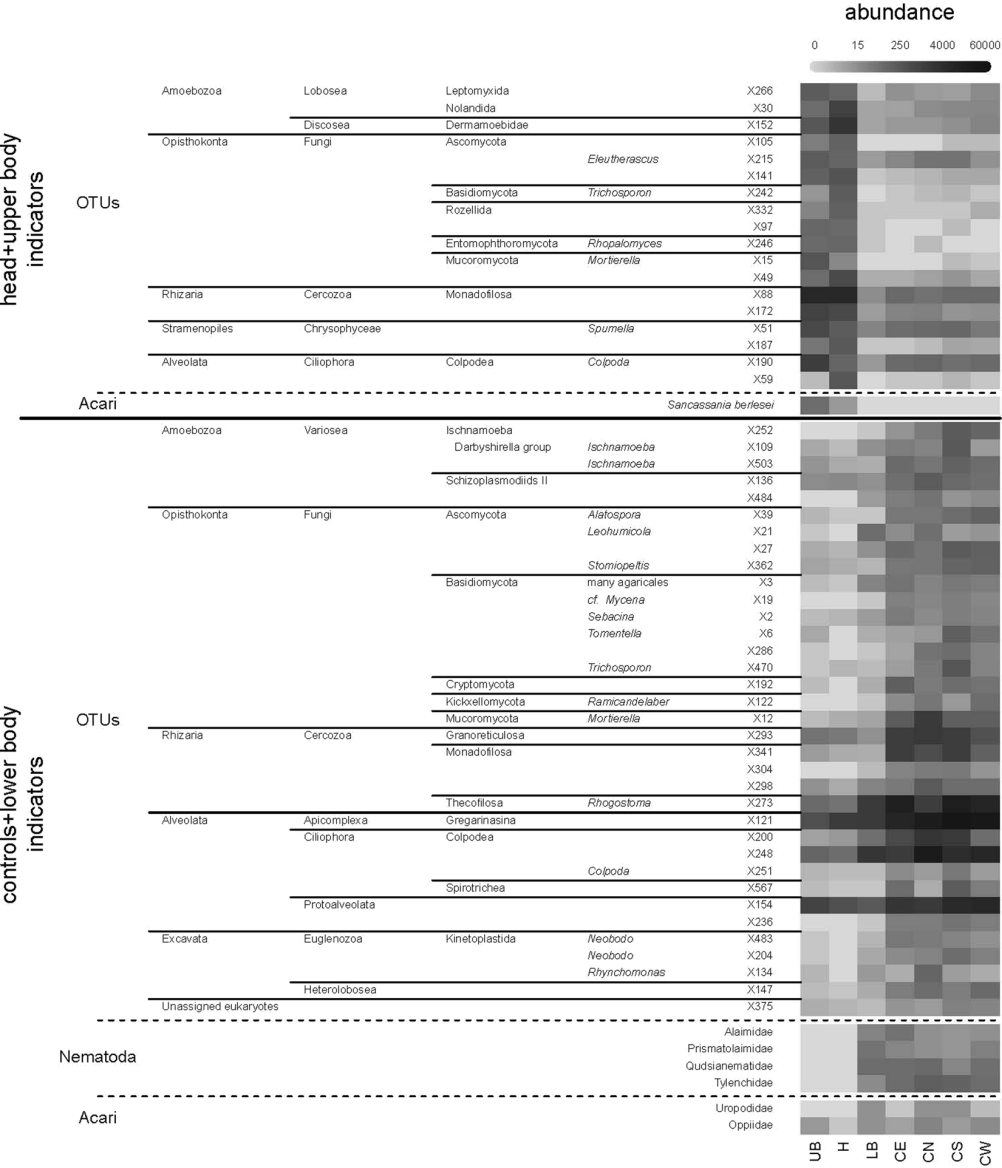
Abundance of the bioindicators of head and upper body (H/UB), and controls and lower body (C/LB) from a criminal case investigation in Switzerland. For each group the eukaryotic bioindicators are sorted according to their taxonomic assignation followed by nematodes families and mite taxa.

The cluster analysis of the community data separated the samples into two groups: 1) head and upper body and 2) controls and lower body (Fig. 2c). In the PCA the sample from beneath the head was separated from all other samples (Fig. 2d). Bacterial feeding Cephalobidae and Myolaimidae were most abundant in the sample beneath the head. The Myolaimidae were only found beneath the head and were further identified as *Myolaimus* sp. (Fig. 2i,j). Tylenchidae (herbivorous), Qudsianematidae (predators, omnivorous) and Prismatolaimidae (bacterivorous) were associated with the controls North and South and the lower body samples (Fig. 2i,j). Control West and East were associated with Teratocephalidae (bacterivorous) and Leptonchidae (fungivorous) (Fig. 2i,j). Control West was associated with the bacterivorous Alaimidae. Four nematode families (Alaimidae, Prismatolaimidae, Qudsianematidae, Tylenchidae) were considered as indicators for controls and lower body (Fig. 3).

### Mites

A total of 391 mites belonging to four Acari orders (Astigmata, Mesostigmata, Prostigmata and Oribatida) were identified from the seven sampling sites. The most diverse and numerous were the Mesostigmata (N = 130; 13 species) followed by the Astigmata (N = 126; five species). A number of soil mites found in the control samples, such as Oribatida and Prostigmata were only discerned as morphotypes (Table 3).

**Table 3:** Total counts of mites from soil samples (500 ml) beneath human remains found in a forest in the Swiss Plateau at approximately 400–450 m above sea level and in control soil samples from the same area.

mites taxa			Head	Upper Body	Lower Body	Control North	Control South	Control West	Control East	total
			H	UB	LB	CN	CS	CW	CE	
Astigmata	Acaridae	*Sancassania berlesei*	13	108	0	0	0	0	0	121
		*Sancassania oudemansi*	1	0	0	0	0	0	0	1
		unidentified sp.	1	0	0	0	0	0	0	1
	Glycyphagidae	*Glycyphagus bicaudatus*	1	0	1	0	0	0	0	2
	Histiostomatidae	unidentified sp.	0	1	0	0	0	0	0	1
Mesostigmata	Gamasida	unidentified sp.	0	2	0	0	0	0	0	2
	Laelapidae	*Stratiolaelaps (Hypoaspis) miles*	3	0	5	0	0	0	0	8
	Macrochelidae	*Macrocheles matrius*	1	0	0	0	0	0	0	1
	Melicharidae	unidentified sp.	0	1	0	0	0	0	0	1
	Pachylaelapidae	*Pachylaelaps longisetus*	2	0	0	0	0	0	0	2
		*Pachylaelaps pectinifer*	0	0	1	0	0	0	0	1
	Parasitidae	*Leptogamasus* sp.	3	0	4	0	0	0	0	7
		*Vulgarogamasus* sp.	4	0	0	16	0	3	0	23
		*Parasitus* sp.	0	0	0	6	0	0	0	6
	Rhodacaridae	*Rhodacarellus* sp.	4	0	0	0	0	0	0	4
	Uropodidae	unidentified sp.	0	0	18	17	17	2	1	55
	Zerconidae	*Prozercon traegardhi*	1	3	0	7	0	0	2	13
		*Mixozercon sellnicki*	5	0	0	0	0	2	0	7
Prostigmata	unidentified	unidentified sp.	0	0	0	11	8	0	0	19
Oribatida	unidentified	unidentified sp.	0	0	0	4	1	5	0	10
	Oppiidae	*Oppiella* sp.	1	12	17	33	11	24	8	106

Most mites were found in the adult stage, with the exception of members of the Astigmata. The Acaridae *Sancassania berlesei* (=*Caloglyphus berlesei*), *S. oudemansi* (=*Caloglyphus oudemansi*), and the Histiostomatidae unidentified sp. 1, 123 specimens in total, were sampled in their immature, phoretic hypopial form. Interestingly, Astigmata were extracted only from the sample sites associated with bones i.e. from the soil beneath the head, upper body and lower body (Table 3). These samples also contained the greatest richness of Mesostigmata.

Considering all seven samples, three Mesostigmata species frequently found in forest soil were the most numerous, the detritivorous Uropodidae unidentified sp. 1 (N = 55), *Prozercon traegardhi* (N = 13) and the predaceous *Vulgarogamasus* sp. (N = 23). A particular soil mite morphospecies of the Oribatida, Oppiidae, *Oppiella* was highly abundant and 100% prevalent.

In terms of the singularity of sample diversity, sample sites and mite species, mites aggregated into three defined clusters. Upper body and head were different from each other and from the remaining five samples, which group together (Fig. 2f). The clustering further indicated that the upper body and head were the two most distinctive samples (Fig. 2e). These two samples contained rare species, which are not typical inhabitants of forest soil. The taxa found in these samples rather correspond to poultry farm or granary species such as *S. berlesei* (hypopi), which is also a predominant species in corpses (especially in advanced decay or mummification stages), accompanied by a specialised predator of these unique farming environments, *Macrocheles matrius* (Fig. 2i,j).

### Micro-eukaryotes

The metabarcoding analysis revealed 386 OTUs from a total of 648′344 sequences, that excluding Metazoa and Embryophyceae (animals and land plants), and rare OTUs (i.e. <0.0005% of the Hellinger transformed community matrix). Globally, the soil micro-eukaryotic community was dominated by fungi, with a majority of Basidiomycota in the control/lower body samples versus a large dominance of Mucoromycotina in the head/upper body samples (Supplementary Figure 1). Likewise, taxa containing many small bacterivores (Heterolobosea, Chrysophyceae, Tubulinea, Cercozoa) were relatively more abundant in head and upper body samples. Variosea (Amoebozoa) as well as the parasitic Apicomplexa were in turn more abundant under the control/lower body samples.

### Multiple Factor Analysis (MFA), hierarchical clustering and Indicator Value

The first dimension showed a clear dichotomy between the head/upper body and control/lower body sample groups (Fig. 2i). This pattern is in line with the separate analyses of the three community matrices (Fig. 2d,f,h) and by the cluster analyses (Fig. 2a,c,e,g). This similarity among results is also shown by the RV coefficients which reveal a significant correlation among the three matrices (Table 4). By contrast, the chemical variables were only correlated with the nematodes data.

**Table 4:** RV coefficients from the multi factor analysis (MFA) calculated on standardized chemical variables and Hellinger-transformed matrices of micro-eukaryotes, nematodes and mites taken from soil samples collected beneath human remains found in a forest in the Swiss Plateau at a height of approx.400–450 m above sea level and in control samples from the same area. The lower left half matrix shows the RV coefficients (in bold) between pairs of matrices while the upper right half matrix (not bold) shows the significance of the corresponding coefficient. “MFA” row and column represent the RV coefficient and p-value between each group of variables and the global model.

RV coefficients	chemical	nematodes	mites	eukaryotes	MFA
chemical components	—	0.0169	0.1297	0.0949	0.0020
nematodes	**0.7741**	—	0.0097	0.0075	0.0123
mites	**0.5774**	**0.8755**	—	0.0103	0.0851
eukaryotes	**0.5938**	**0.8949**	**0.9446**	—	0.0613
MFA	**0.8036**	**0.9709**	**0.9329**	**0.9421**	—

A total of 97 variables (three chemical variables, 79 eukaryotic OTUs, seven mite taxa and eight nematode families) were characteristic of the first dimension of the MFA ordination (Fig. 2j). From the 97 variables characteristic of the first dimension, the majority were well associated with the head/upper body samples (3/3 chemical variables, 61% of micro-eukaryotic OTUs, 2/8 nematodes families and 86% of mite taxa).

The IndVal analyses revealed 18 eukaryotic OTUs and the mite species *Sancassania berlesei* as head/upper body indicators, while indicators for controls/lower body indicators, included 53 eukaryotic OTUs, four nematode families (Alaimidae, Prismatolaimidae, Qudsianematidae, Tylenchidae) and two mite families (Uropodidae, Oppiidae) (Fig. 3).

## Discussion

The analyses of this case were divided into two parts: the examination of the human remains alone and the analyses of the soil samples beneath the remains.

The PMI estimation via bomb peak radiocarbon dating resulted in a time range of up to 3 years (hair F^14^C-data). From the anthropological investigation, it was also assumed that the bones could not have been lying outside for less than one year due to the lack of fatty appearance, no filling of the medullary cavity of the femur, no smell and no soft tissue^36^ except of hair, which was found right beneath the occipital bone (*Os occipetale*). Although changes in taphonomy, in particular a faster decomposition due to the thermal destruction (fire) cannot be excluded. In conclusion, radiocarbon dating and anthropological analysis suggest that the individual must have been died between one to three years before the remains were found.

When interpreting the F^14^C-data of the collagen, the turnover rates of human bones were based on previous studies^8,37–39^. Since this individual was a late adolescence or young adult male at the time of death (based on anthropological bone features), a turnover rate of 10–30% per year (between the age of 10 and 15 years) and 3–1.5% per year (after the age of 20 years) has to be considered^38^. Therefore, collagen data are always mixed values containing the carbon signal of the last years of life. Nevertheless, the collagen of the femur has its core area between 9–12 years before the remains were found. Assuming that most of the incorporated carbon derives from the two first life decades the data are compatible with a young adult.

The individual was subsequently identified via a DNA match in the EDNAIS database, Switzerland. A witness statement showed that the individual was last seen 22 months before the remains were found (summer/date under nondisclosure). All data are thus concordant with this information.

The analyses of soil samples beneath decomposing cadavers has generally observed a sharp increase of soil nutrient content and pH during the active and advanced decay phases of cadaver decomposition^40^. This is when the most rapid breakdown of a cadaver takes place^14^. In this study, bones were found at the stage of dry remains, which does not mean that the concentration of nutrients have returned to basal levels^14^. According to the marker categories we have developed^41^, pH and NH_4_^+^ are described as early peak markers (EPMs), showing significant increases at the beginning of the decomposition process (Szelecz *et al*. submitted). The absence of these early peak markers in the samples beneath the remains suggests two interpretations, either the timespan since death was sufficient for EPMs to return to basal levels or the body had started decomposing elsewhere and was transported to the find site when it had already reached a later decomposition stage i.e. advanced decomposition.

At later decomposition stages, Ca^2+^ and other additional late peak markers (LPMs) such as NO_3_^−^ increase^15,42^. NO_3_^−^ levels were significantly elevated beneath carcasses one year post-mortem^42^ which was confirmed by our studies^41^. The lack of higher NO_3_^−^ concentration under the cadaver in this study suggests that the PMI was >1 year. The absence of LPMs such as Ca^2+^ might indicate an upper limit for the PMI as shown by Melis *et al*.^15^ who observed elevated calcium levels at carcass sites from three to six years post-mortem probably released from the bones. However, patterns of soil nutrient response to decomposing cadavers vary among studies^15^ and thus it is currently not possible to develop a precise PMI estimation method from soil chemical characteristics alone.

The presence of elevated levels for P (head and upper body) and N (head) indicate that the time elapsed since peak decomposition was not long enough for these markers to return to basal levels. Significantly elevated P levels were described one and three years post-mortem^16^. N levels beneath large ungulate carcasses were significantly higher two years post-mortem and 10 times higher than in the surrounding soil even three years post-mortem (not significant)^16^. Nevertheless, elevated levels of P beneath the head and upper body, plus a higher N concentration beneath the head and the clustering of head and upper body samples suggests that the decomposition process had at least partly taken place on site. In contrast, the lower body part groups with the controls indicate that decomposition may have been hindered or not taken place in this area. This is in accordance with the findings that the lower body parts were burnt much more than the upper body parts (excluding arms). However, this interpretation should be taken cautiously given the sample size of our study.

Nematode density in all samples was within the range reported elsewhere for terrestrial nematodes^26,43^ and the three most abundant families that occurred in all samples i.e. Cephalobidae, Rhabditidae and Plectidae are common in soil^44^. The clustering grouped nematode communities from controls and lower body samples together and revealed four indicators: Alaimidae, Prismatolaimidae, Qudsianematidae and Tylenchidae. Nematodes can be classified according to their feeding habits and life-history characteristics^43,45^. Among the indicators Alaimidae and Qudsianematidae are typical K-strategists (persisters) with a high sensitivity to disturbances^46^. Tylenchidae are very tolerant to disturbances, they are r-strategists (colonizers) and frequently found in soils^44^. Prismatolaimidae can be considered as intermediate between these two groups being more sensitive than Tylenchidae^46^. The presence of nematodes that are sensitive to disturbances in line with a higher family richness indicates that the control and lower body samples might not have been exposed to stress^26^.

By contrast, head and upper body samples were characterized by a low family richness and were dominated by r-strategists i.e. bacterial feeding nematodes that are tolerant to pollutants and organic matter decomposition^46^. Especially Myolaimidae which only occur in the head samples were shown to be enrichment opportunists^47^. Myolaimidae were further determined as *Myolaimus* sp., which is rarely recorded in samples from terrestrial habitats^48^. During the composting process *Myolaimus* sp. was restricted to the last phase of the composting process (maturation) indicating that it might have special requirements^49^. Therefore, *Myolaimus* sp. might be an indicator for a late decomposition stage suggesting that only part of the decomposition process took place *in-situ* and bones and some flesh remained after the fire to decompose. Nevertheless, in a study on the effect of decomposing pig cadavers on soil nematode communities in the same general region (albeit in a different forest and soil type) Myolaimidae did not occur at all during the whole decomposition process within a one-year post-mortem period^26^. In that study, it was also shown that family richness was still significantly lower 263 days post-mortem^26^. Here further studies are necessary. Bearing in mind that comparable data are sparse, a PMI of at least 8–9 months but more likely >1 year is suggested based on the nematode data.

Of the seven samples analysed in this study two stand out due to their unique mite fauna composition, which is exogenous to the forest soil and comprised species of forensic importance (previously found on corpses) and used as trace evidence. Communities from the upper body, followed by head are unique in that they contain the only and highest number of *Sancassania berlesei*, and the only specimens of *Macrocheles matrius*. Both, *S. berlesei* and its main predator, *M. matrius* are inhabitants of more synanthropic habitats, particularly those related to granaries, farms (poultry and pig hay-beddings) and less often agricultural lands (cereal fields). In Europe, these species are found in compost, poultry litter, decayed bulbs and tubers, especially deep litter broiler houses, and have been originally found in stored grains or food products^50–52^. Interestingly, *S. oudemansi* (found in the head sample) is even more restricted to synanthropic habitats, poultry settlings or granaries, wherever wheat or other grains abound^51–54^.

The large numbers of *Sancassania* all found in hypopial stages, in upper (the body and head) samples indicate: 1) that the human remains and their associated *Sancassania* (and *Macrocheles*) mites have originated in one of the aforementioned habitats (e.g. a farm); 2) that a massive *Sancassania* population was living from the remains; and 3) that this well-developed population was suddenly exposed to very unfavourable environmental conditions (e.g. drought, fire). These facts are strong indicators of movement or relocation of the remains. From the data of the two key sites (upper body and head) it is possible to infer that the original massive mite population very likely built up from (early) active to advance decay, and that at this stage the remains were transported and deposited in their final resting place, the forest soil, more precisely on a limited space or position where the upper body and head bones were later found. This is also explained by the lack of any other forensic markers, characteristic of decay on forest soil. Indeed, there is no acarological evidence indicative of decomposition having taken place on the forest soil.

*Sancassania berlesei*, like the majority of stored-product mites (Astigmata) are sensitive to humidity; exposure to low humidity levels leads either to death or to moulting into more resistant immature stages commonly known as hypopi^51,54,55^. Optimal conditions imply very high humidity levels; water saturated habitats or exceptionally damp conditions, such as enclosed or sealed habitats. A good example would be a human corpse wrapped, concealed in clothes, inside bags or inside any sort of sealed container able to maintain the damp conditions needed for the mites to thrive^1,27,30,56–60^. This might explain the well-developed colony of *S. berlesei* found only in a reduced area or particular patch of soil (upper body and head sites). Soil under the lower body bones did not contain any forensically relevant mite species. Mites in this area are the same as those found in controls, lower body and clustered together with most controls. This suggests that the lower body site was not the place or location of the ‘container’ or ‘bag’ with human remains, and perhaps the bones observed on this area were the product of the movement of the bones by forest scavengers (foxes, dogs, birds, etc.). Under unfavourable conditions such as drought and lack of food, or food lacking nutritional content, *S. berlesei* will always produce hypopi and most of these hypopi are active forms. Hypopi are second instar nymphs able to disperse mainly (not exclusive) as phoretic on other animals to new, optimal environmental conditions^30,51,61,62^.

**Figure 4:**
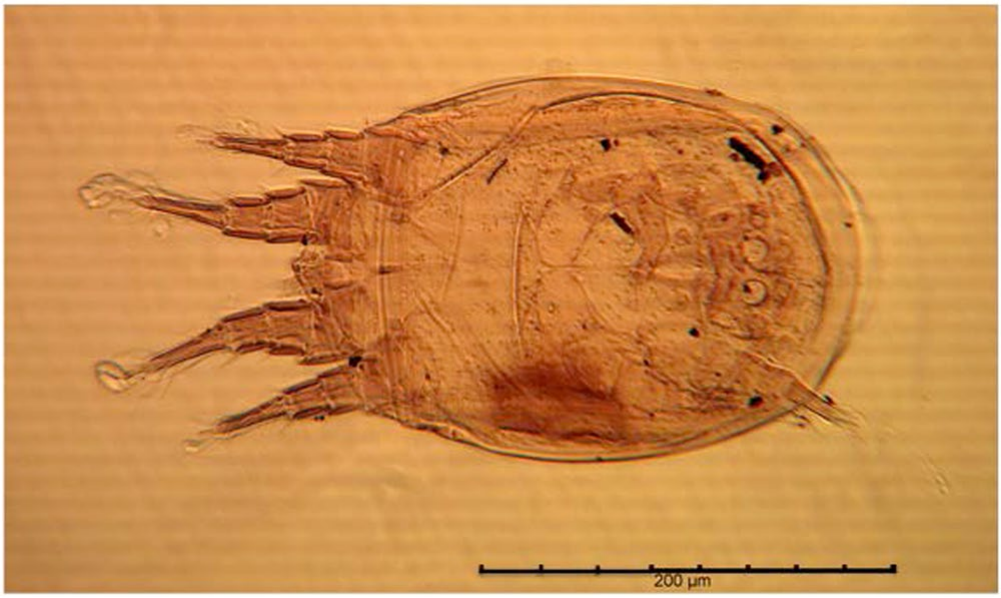
A hypopus of *Sancassania berlesei* from the head sample site (H) from a criminal case investigation in Switzerland.

Only a few insects have been described as hosts of *S. berlesei* hypopi, particularly Scarabaeidae (chaffers) and Tenebrioniidae (stored product beetles)^62–64^. The unique association with the flour beetle, *Tenebrio molitor*, confirms its habitat specificity to granaries or stored grain facilities. Termination of hypopial forms will occur when both high humidity and food are restored, moulting to the next stage or trinonymph; otherwise they will die as hypopi^55^. Experiments designed to produce hypopi considered exposure to dry cultures and lack of food^55^. All 121 *S. berlesei* collected in this case were hypopi (Fig. 4), no adult was found. It means that the massive population went through a bottleneck of unfavourable conditions, like exposure to extreme drought or lack of food. In addition to mites, the two sample sites upper body and head contained the majority of charcoal particles, indicative of fire and the site where the remains might have been burnt. The remains must have been burned exactly in that patch of forest soil. The fire most likely consumed the external layers of the wrapped remains killing most mites of the outer parts. The thickness of the massive population of perhaps thousands to millions of mites, which probably reached several cm, protected the most internal part of the colony from fire. Those hundreds of *S. berlesei* that survived the fire were then exposed to a foreign environment with no food, not enough humidity, produced resistant offspring or hypopi while surrounded by soil predators.

Remarkably, the presence of a second mite species, a strict inhabitant of farm/granary habitats affirms the origin of the fauna associated with upper body and head. *Macrocheles matrius* is a predator, a foreign species to the forest soil and to carcasses/corpses, but specialized in hunting and consuming the acarids of granaries or poultry litter^50,54,65^. In terms of PMI estimation, the massive colony of mites inhabiting the original human remains that reached the forest soil are similar to that of a concealed corpse in its later stage of decomposition, very likely a mummified corpse. Judging by the size of the surviving population of *Sancassania* hypopi, and the time it takes to mummification of a human adult inside a sealed enclosure, it is possible that death and concealment of the body happened at least 8–12 months before the remains were disposed on the forest soil. This is due to the life-span of these hypopi which might have struggled to survive in the new habitat and conditions. Mite traces are good indicators of what might have happened from the moment of death until decomposition reached advanced stages, in this case indicating the corpse location on the forest soil, perhaps its origin (location of death) and confirming the burning process.

The presence of a decomposing corpse has clearly modified the micro-eukaryote communities. The combination of our two statistical analyses (IndVal and MFA) allowed us to determine a limited number of OTUs that were either typical for (1) head and upper body (H/UB) samples or (2) controls and lower body (C/LB).

General communities differ considerably between head/upper body samples and control/lower body. Basidiomycetes, which dominated the control and lower body samples, are virtually replaced by Mucoromycota in the head and upper body samples. While the first group includes many saprotrophs and mycorrhizae, the second includes typically r-strategists such as *Mortierella* spp. This shift has been observed also in the case of perturbations such as disruption of connections between the root and the fungus^66^. The higher incidence of mainly bacterivorous taxa is associated with higher values of P and N, which are expected to increase bacterial densities.

Soil fungi are the best-studied group of soil micro-eukaryotes and thus their community patterns are easy to interpret. For instance, OUT X242 is affiliated with genus *Trichosporon*, a fungus which belongs to the normal skin microbiota^67^. Members of this genus have been found associated with late stages of decomposition of human bodies^68^. OUT X246 (*Rhopalomyces*) is an exclusive parasite of nematode eggs^69^; its abundance in the head and upper body samples is thus consistent with the high number of nematodes found in these samples. Likewise, the prevalent presence of ectomycorrhizal fungi among the controls and lower body (C/LB) indicators (i.e. OTUs X3, representing several tree mycorrhizae in forests and X362, a Pezizomycotina affiliated to genus *Stomiopeltis*) was to be expected as these organisms are ubiquitous in forest soils.

Several species of amoeboid protists, known to occur only in stable systems, were found amongst the indicator OTUs of the controls and lower body samples. Amongst them, Variosea (Amoebozoa) from the Ischnamoeba/Darbyshirella clade (X109, X503) are reticulate organisms with very thin and delicate pseudopodia which are typical slow-growing organisms^70^. *Rhogostoma* (X273), like many other testate amoebae are also typically K-selected organisms that are supposed to perish underneath cadavers^24,25^. On the other hand, the presence of OUT X152, affiliated to the amoebozoan genus *Mycamoeba* suggests a beginning of recovery, as these organisms were shown to be negatively influenced by the presence of a cadaver in an experimental setup containing three pig corpses laid on forest soils^71^. The presence of this particular OTU therefore suggests a PMI of over one year^71^.

## Conclusions

Following the analysis of five evidential components the following course of events can be proposed for this case: Human remains were found in a Swiss Plateau forest. The examination of the bones and the hair revealed that the remains were from a young-adult human male who must have died 1–2 years before the discovery. This person was subsequently identified and was last seen 22 months before his remains were found. The bones showed signs of scavenging and exposure to fire, likely the remains were burnt *in-situ*. The analyses of chemical constituents, nematodes and micro-eukaryotes revealed that the decomposing remains had probably been on the site for at least 8–9 months and more likely more than one year. This means at least part of the decomposition process had taken place on site.

Mite evidence, however, suggests that the corpse was translocated from its original crime scene to the forest soil. It first decomposed *in-situ*, reaching late stages of decomposition before being moved to the forest. According to survival rates of *Sancassania* hypopi, the remains were likely burnt in a small patch on the forest soil, just a few months before discovery.

**Figure 5:**
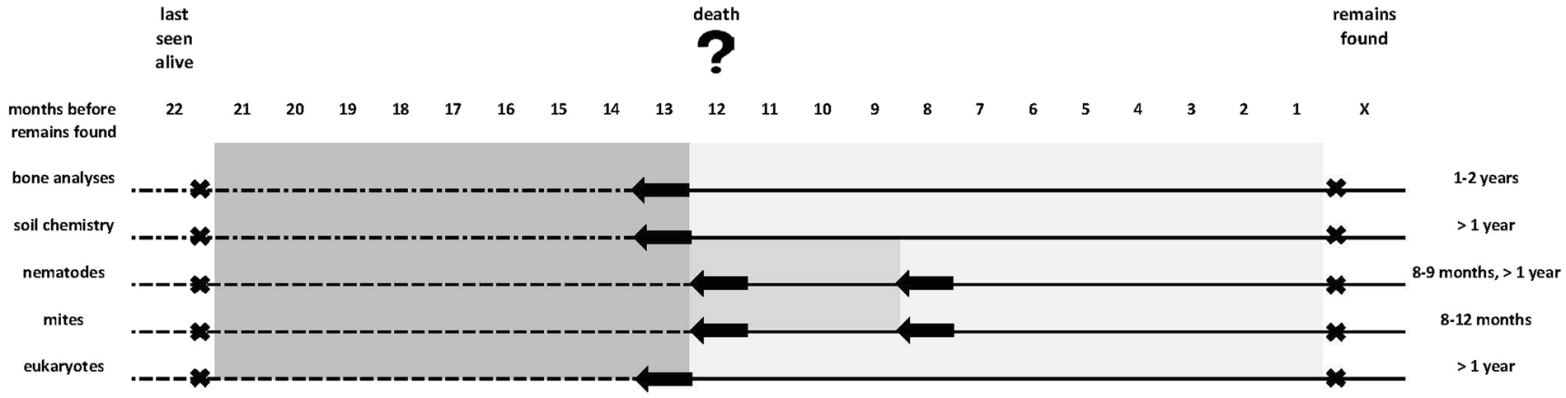
Reconstruction of the body posture and soil sampling area.

If the decomposing remains were transported to the site at least 12 months before the finding, the person must have died between summer and spring before that. Bearing in mind that decomposition is accelerated by temperature and insect access to the body^10,72^ no tissue would have been left if the person had started to decompose at a different location in summer. Instead, our findings suggest that the person was killed probably in autumn or winter, the corpse started to decompose in a confined environment, possibly a farm (or similar), and was perhaps brought to the final or finding site in the early spring of the following year where it was partially burned (Fig. 5).

Although some of the methods shown are still in the process of being developed (e.g. nematodes as forensic indicators for PMI estimation), others are already well-established (e.g. forensic anthropology). Nevertheless, the interpretation of a crime scene gathered from the simultaneous study and combined analysis of independent lines of evidence illustrates the added value of this multiproxy approach (Fig. 5). The main aim of this work was to provide a strong incentive for case work as well as experimental studies to further develop a comprehensive toolbox for forensic or crime scene investigations.

## Material and Methods

### Case history and sampling

**Figure 6:**
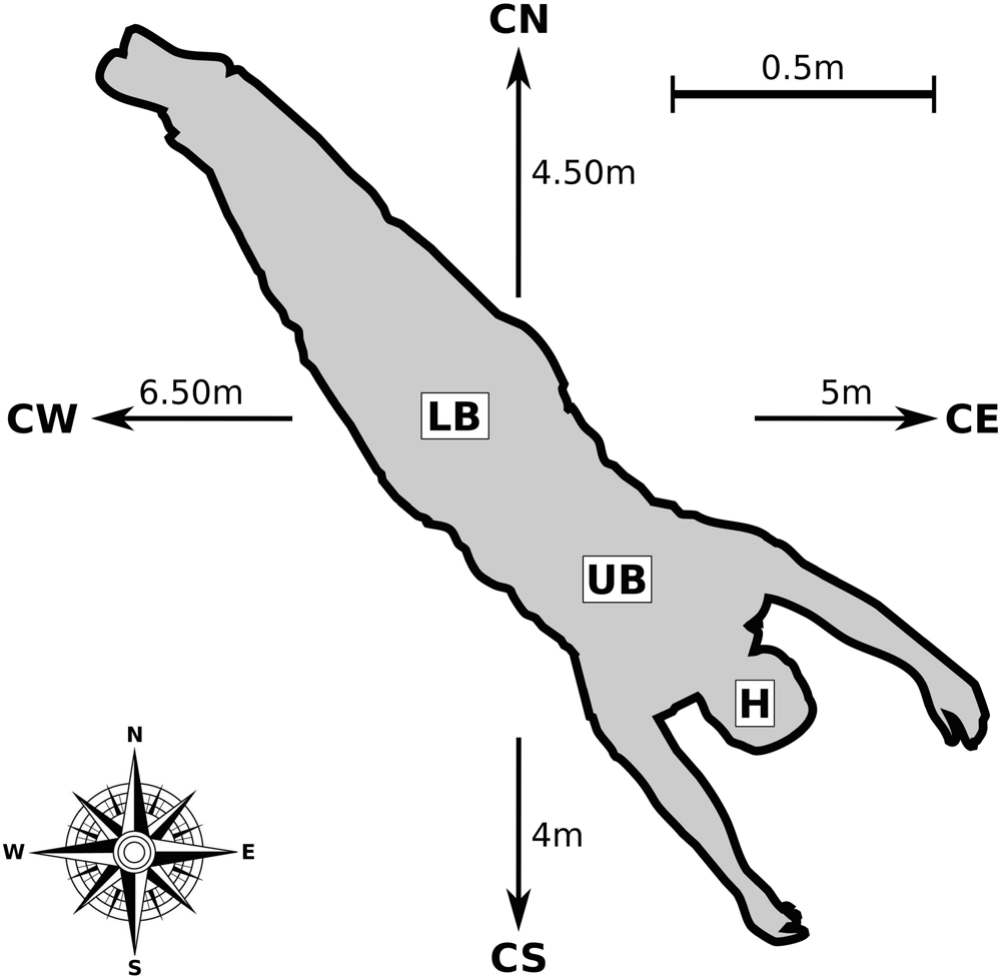
Summary timeline of all suggested PMIs from a criminal case investigation in Switzerland.

Human bones were found in a forest in the Swiss Plateau at a height of approx. 400–450 m above sea level. The ground was covered with dry leaves and the bones were partly covered with leaves and small branches. After their removal, the bones showed signs of thermal destruction. Some burned tree trunks with a diameter of approx. 20–30 cm and bigger branches were arranged in a square approximately 2 × 2.5 m around the bones. The branches originally covering the bones showed signs of charring and had been removed by the finder. These burnt branches were partially overgrown by moss. Due to the position of the skeletal elements in comparison with their biological anatomy, the location of the body before decomposition and burning, could be reconstructed (Fig. 6). Beside the bones, some scalp hair appeared below the *Os occipetale* during the recovery and residues of different fabrics, a key, some coins and jewellery were found.

Soil samples (soil cores of 10 cm in diameter by 10 cm depth) were taken from the area where the bones were found i.e. beneath the head (H), upper body (UB) and lower body (LB) and from control areas not impacted by the cadaver or the fire in the four cardinal directions, 4–6.5 m from the body: North (CN), South (CS), East (CE) and West (CW) (Fig. 6). The surrounding area was searched for further bones and everything was transferred to the Department of Physical Anthropology, Institute of Forensic Medicine, University of Bern, for examination.

### Bone and hair analyses

The human bones which did not show severe thermal destruction were investigated anthropologically according to Acsádi and Nemeskéri^73^ (1970), Buikstra and Ubelaker^74^ (1994) and Rösing *et al*.^75^.

A 5.1 gram sample of the femoral diaphysis without any visible thermal destruction was sent for radiocarbon dating to CEZ Archäometie gGmbH, Mannheim, Germany. Additionally, 2.0 gram of hair was also sent for radiocarbon dating in order to perform a bomb peak application^8,39^. Bone collagen was extracted^76,77^ and the hair was carefully cleaned, both were measured via MICADAS-accelerator. The calibration was performed with CALIBomb^78^.

### Soil chemical analyses

Soil pH was measured in the laboratory with a pH metre after diluting the sample in water in a 1:2.5 proportion. Ammonium and nitrate analyses were performed directly after sampling using colorimetric determination^79^. Total carbon and nitrogen was determined using a CHN analyser (Thermo Finnigan Flash EA 1112) on dry, ground soil. Bioavailable phosphorus content was determined by colorimetric analysis according to the Olsen method^80^. The concentrations of Mg^2+^ and Ca^2+^ were determined using inductively coupled plasma optical emission spectrometry (Perkin-Elmer Optima 3300 DV ICP-OES) preceded by a cation exchange capacity extraction (CEC, Cobaltihexamine method). Potential chemical soil markers were classified into (1) early peak markers (EPM) that show the highest concentrations early in decomposition until advanced decay following degradation to (2) late peak markers (LPM), during the dry and remains stage. Some of them might show (3) late elevated levels (LEL)^41^.

### Nematodes

Nematodes were extracted from 100 g of soil using a modified Baermann funnel technique according to the protocol of Brown and Boag^81^. Nematodes were counted alive using a dissecting microscope (Olympus SZ51) and then fixed with heated formaldehyde (4%) and heat-killed at 65 °C for 3 minutes. One hundred randomly chosen nematodes per sample were identified to family level using an upright light microscope at 400x magnification (Axio Lab.A1, Zeiss). Identification was based on the identification guides of Bongers^82^ and Scholze and Sudhaus^83^. All nematode densities are given in individuals per 100 gdw.

### Mites

A 500 mL soil sample was collected from each sampling site and placed on Tullgreen-Berlese funnels to extract moving mites into a collector jar with 70% ethanol (following the method used by Saloña *et al*.^60^). Mites were cleaned from the collector jars, cleared for permanent mounting in Hoyer^84^ and identified using a variety of keys and descriptions^51,65,85–89^. A voucher number was assigned to all microscope slides and they were deposited in the Forensic Acarology Reference Collection, Acarology Lab, University of Reading.

### Soil micro-eukaryotes

Total soil DNA was extracted using the MoBio PowerSoil isolation kit following the manufacturer’s instruction. To screen the eukaryotic diversity of the seven soil samples, the V9 fragments of the small sub-unit ribosomal RNA (SSU rRNA) was amplified following the protocol of Amaral-Zettler *et al*.^90^ and the amplicons sequenced with an Illumina HiSeq. 2000® sequencer (Fasteris, Geneva, Switzerland). The resulting V9 sequences were then processed as follows: Sequences were removed when the average phred score of a 50 nucleotides window was below 20. Chimera were identified and discarded using the program Uchime (v. 7.0.1090)^91^ by comparing sequences among them and to the PR^2^ database^92^. The sequences were then clustered into Operational Taxonomic Units (OTUs) using the software Swarm (v. 1.2.12)^93^ with the default parameters. The OTUs were finally taxonomically assigned by aligning the dominant sequence of each OTU to the PR^2^ database using the program Ggsearch36 (Fasta package v. 36.3.6 http://faculty.virginia.edu/wrpearson/fasta/CURRENT/) with the default parameters. OTUs assigned to prokaryotes were removed from the analysis as well as those assigned to Metazoa or Embryophyceae in order to avoid bias which would be caused by a piece of those macro-organisms in the soil sample.

### Numerical analysis

Chemical variables were scaled and community matrices (Eukaryotes, nematodes and mites) were Hellinger-transformed^94^. As metabarcoding data are prone to yield a huge proportion of rare OTUs, we discarded OTUs representing less than 0.5‰ of the Hellinger-transformed matrix. A multiple factor analysis (MFA) (package FactoMineR v. 1.31.4^95^), was then computed with the four data matrices to assess the correlative structure of the data. A hierarchical clustering was also performed with Euclidean distances on the chemical variables and with Bray-Curtis distances on each of the three community matrices.

The bioindicator value of each taxon or OTU in each of the three community data sets was assessed using an indicator species analysis (function indval; package labdsv 1.6–1)^96^. For this analysis, we used two groups based on the results of the four clusterings and the first dimension of the MFA: (1) head and upper body, and (2) lower body and controls. OTUs were selected as bioindicators if the p-value of their indicator value was under 0.05 after 10000 iterations. As the number of OTUs was high, many were significant in the IndVal analysis. To keep the number manageable, we only retained those that had both a significant Indval and showed a clear pattern in the first dimension of the MFA.

### Data availability statement

Due to legal reasons (criminal case investigation), the availability of the data is restricted and data must be kept under nondisclosure.

## Electronic supplementary material

Supplementary Information
