# Supplementary material for: Comparative analysis of bones, mites, soil chemistry, nematodes and soil micro-eukaryotes from a suspected homicide to estimate the post-mortem interval
Source: Sci Rep. 2018 Jan 8;8:25. doi: 10.1038/s41598-017-18179-z (PMC5758714; doi:10.1038/s41598-017-18179-z)
Supplement: Supplementary file 1 — Supplementary Information [file 41598_2017_18179_MOESM1_ESM.pdf]

Comparative analysis of bones, mites, soil chemistry, nematodes and soil micro-eukaryotes from a suspected homicide to estimate the post-mortem interval

Ildikó Szelecz, Sandra Lösch, Christophe V.W. Seppey, Enrique Lara, David Singer, Franziska Sorge, Joelle Tschui, M. Alejandra Perotti, Edward A.D. Mitchell

Supplementary Material Figure 1. Relative abundance of taxon sequence abundances (micro-eukaryotes) from head and upper body (H/UB), and controls and lower body (C/LB) from a criminal case investigation in Switzerland. Only taxa representing at least 1% of the total abundance are shown.

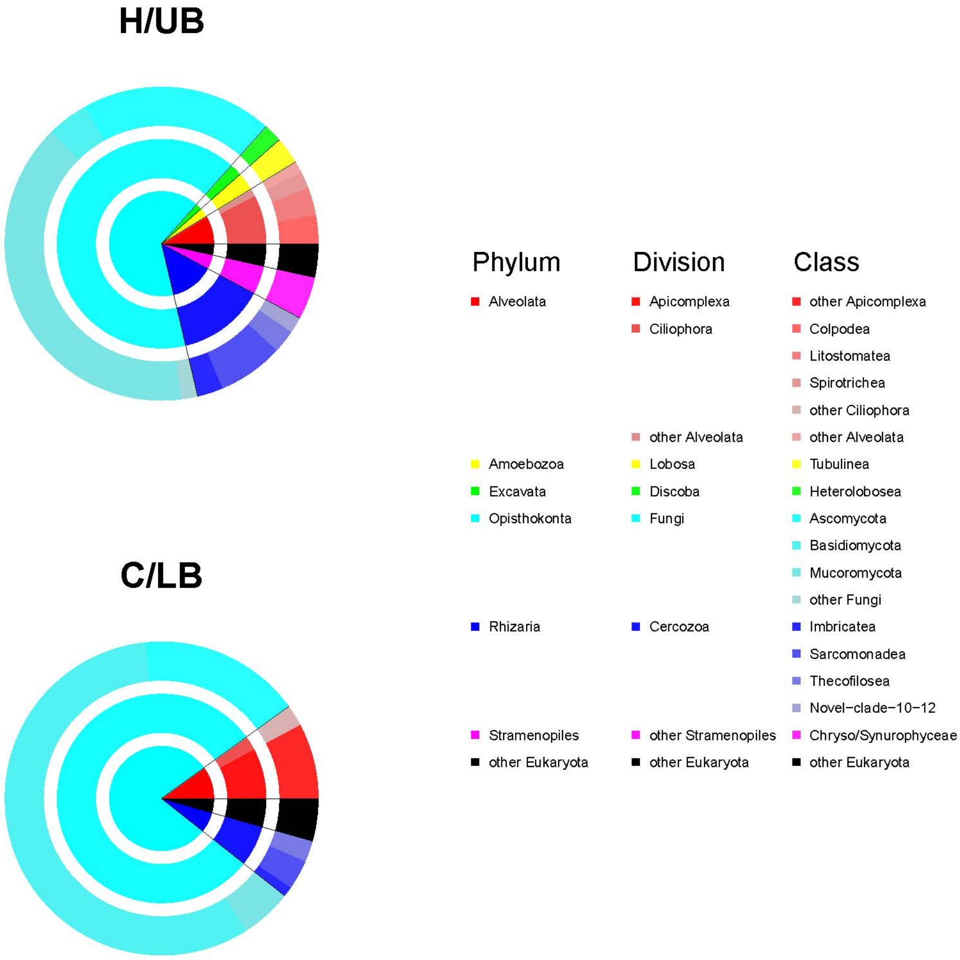

H/UB
C/LB
Phylum
Division
Class
Alveolata
Apicomplexa
other Apicomplexa
Ciliophora
Colpodea
Litostomatea
Spirotrichea
other Ciliophora
other Alveolata
other Alveolata
Amoebozoa
Lobosa
Tubulinea
Excavata
Discoba
Heterolobosea
Opisthokonta
Fungi
Ascomycota
Basidiomycota
Mucoromycota
other Fungi
Rhizaria
Cercozoa
Imbricatea
Sarcomonadea
Thecofilosea
Novel−clade−10−12
Stramenopiles
other Stramenopiles
Chryso/Synurophyceae
other Eukaryota
other Eukaryota
other Eukaryota
